# Fully Defined 3D Hybrid System for Bone Tissue Engineering: Integration of MeHA–RGD/PCL–TCP Scaffolds With Human Stem Cells via 3D-Printed Vacuum-Assisted Cell Loading Device

**DOI:** 10.1155/term/7287217

**Published:** 2025-07-03

**Authors:** Jolene Quek, Catarina Vizetto-Duarte, Kee Woei Ng, Swee Hin Teoh, Yen Choo

**Affiliations:** ^1^Developmental Biology and Regenerative Medicine Programme, Lee Kong Chian School of Medicine, Nanyang Technological University, Singapore 308232, Singapore; ^2^School of Materials Science and Engineering, Nanyang Technological University, Singapore 639798, Singapore; ^3^Nanyang Environment and Water Research Institute, Nanyang Technological University, Singapore 637141, Singapore; ^4^Centre for Advanced Medical Engineering, College of Materials Science and Engineering, Hunan University, Changsha 410012, China

## Abstract

Despite ongoing efforts, the regeneration of critical-sized bone defects remains a significant challenge for clinicians due to the absence of a standard clinically compliant bone tissue engineering protocol. These challenges are mostly attributed to the inadequacies of current methods, characterized by their high variability and the reliance on animal-derived components, such as fetal bovine serum (FBS) in cell culture. To address these shortcomings, our approach diverges from conventional practices by prioritizing consistency and reproducibility, and the complete elimination of animal derivatives throughout the entire process. We have developed a novel method that utilizes a peptide-functionalized photocrosslinkable methacrylated hyaluronic acid (MeHA–RGD) hydrogel as a cell sealant for loading human adipose-derived stem cells (hASCs) into a 3D porous polycaprolactone–tricalcium phosphate (PCL–TCP) scaffold. Additionally, we created a 3D-printed vacuum-assisted cell loading device to facilitate this process and ensure efficiency and consistency during cell loading. Our findings indicate that the MeHA–RGD hydrogel supports both stem cell viability and osteogenic differentiation, demonstrating outcomes comparable to those achieved with fibrin glue, a conventional cell sealant widely used in BTE from autologous or xenogeneic sources, even under serum- and xeno-free conditions. In the pursuit of clinical translation, it is vital that biomaterials exhibit low variability, are easily accessible, readily available, and completely free of animal derivatives. To our knowledge, this is the first study to employ a 3D-printed vacuum-assisted cell loading device system within a fully defined hybrid 3D system under complete serum- and xeno-free conditions. These findings unravel and encourage alternative approaches in addressing the existing challenges in BTE, thereby facilitating and accelerating clinical translation in the future.

## 1. Introduction

Almost 2.2 million bone graft surgeries are performed annually [1, 2], making bone the second most transplanted tissue after blood [3]. Bone inherently possesses excellent self-healing ability; however, large bone defects caused by infection, tumors, and trauma are unable to spontaneously heal [4] and require large volumes of bone graft, making the treatment of large segmental bone defects a clinical challenge for orthopedic and plastic surgeons [5–7]. Various strategies are employed to reconstruct large segmental bone defects, with autografts considered the gold standard [8, 9]. Autografts involve transplanting bone from the patient's own body but are limited by donor-site morbidity, postoperative complications, and restricted tissue availability for critically sized defects [10]. Allografts, derived from donors or cadavers, address some limitations of autografts, offering greater availability without the need for additional surgery [11]. However, they exhibit reduced osteogenicity and mechanical strength, as well as delayed osteointegration, often failing at the host–graft junction. Additionally, allografts carry risks of rejection and disease transmission [8, 12]. Thus, while autografts and allografts have shown clinical success, their drawbacks—including morbidity, limited volume, and infection risks—make them insufficient for treating critical-sized bone defects [13]. Bone tissue engineering (BTE) has emerged as a promising alternative for fracture management compared to the use of traditional bone grafts, as it eliminates the need for bone donation [14]. Tissue engineering therapeutics usually comprise several components, including stem cells, biocompatible scaffolds, morphogenic signals for cells to adhere and differentiate into the desired phenotype, and sufficient vascularization for nutrient and oxygen supply [8]. Scaffolds act as a structural framework, supporting cell attachment, proliferation, and organization. Morphogenic signals, such as growth factors and cytokines, play a critical role in guiding stem cell differentiation into the desired phenotype. Effective vascularization within the engineered construct is vital for delivering nutrients and oxygen while removing metabolic waste, ensuring cell functionality and viability [15]. Addressing large segmental bone defects remains particularly challenging due to their compromised wound healing environment, inadequate vascularization, and the requirement for large-volume bone regeneration to achieve successful healing [5].

Despite numerous innovations in BTE and successful outcomes in preclinical studies of both small and large animal models, along with several clinical studies [16–19], at present, there is no routine clinical protocol for bone regeneration using BTE [20–24]. Conventional BTE approaches have several drawbacks, mostly involving highly variable components or experimental setups (such as manual cell loading into the scaffold), and the use of animal-derived compounds and biological components (such as fetal bovine serum (FBS) and fibrin glue). These factors skew experimental results, making it challenging to achieve reproducible results and impeding routine clinical translation.

Effective cell delivery into scaffold constructs plays a vital role in the success of functional bone tissue regeneration. Low cell seeding efficiency, inhomogeneity [25], and poor cellular attachment can lead to heterogeneous mechanical and biological properties of the engineered bone tissue, resulting in poor bone quality [26–28]. The initial seeding density can affect the overall expression of osteogenic genes in the 3D construct: At low cell densities, cellular contact is minimized, compromising bone formation, whereas, at extremely high cell densities, there is limited nutrient transport and waste removal, thereby compromising cellular viability and differentiation [29, 30]. Critical-sized defects require a much larger volume of the implant, which can be challenging to load homogeneously [31, 32]. Currently, cells are typically seeded into the scaffold by resuspending the cells in a hydrogel and loaded using manual pipetting, or alternatively simply cultured with the scaffold in expansion media to allow cell adherence over time [33–36]. Consequently, controlling cell attachment and distribution within the scaffold is difficult, limiting the quality of bone constructs in BTE.

Fibrin glue is frequently employed as a sealant for cell attachment and delivery into scaffolds [37, 38]. Fibrin glue is a two-part adhesive comprising fibrinogen and thrombin. It is available from human allogeneic pooled or single-donor blood donors, autologous blood, or animal sources. Commercial sources such as Tisseel (Baxter), Beriplast P (Behring), Tissucol (Baxter), Green Plast (Orleant), and Cryoseal (ThermoGenesis) offer the advantage of known compositions and ease of use. Commercial fibrin glue comes in fixed concentrations, typically with thrombin activity reported at 4–1000 IU/mL and fibrinogen concentration at 20–100 mg/mL [39–41]. However, when derived from xenogeneic sources, there is a risk of interspecies contamination and potential immunological reaction against xenogeneic serum antigens [42]. The alternative approach is to prepare fibrin glue directly from the patient [43]. However, composition and quality variations are influenced by the patient's gender, age, and health status [44], with preparation methods also impacting the recovered fibrinogen and thrombin concentrations [45]. Moreover, fibrin glue presents challenges such as uncontrollable clotting, rapid degradation, labor-intensive preparation [46], lengthy setting time [47], and high costs [48, 49]. Despite its widespread use as a biomaterial in clinical and research applications, fibrin glue has several drawbacks that limit its clinical utility. These include rapid degradation in vitro and in vivo, poorly understood shrinkage behavior, weak mechanical properties, and significant batch-to-batch variability [50].

Another limiting factor for the successful clinical translation of BTE for critical-sized bone defects is the presence of xenogeneic components in the cell manufacturing process, such as FBS used in the in vitro expansion and differentiation of stem cells. FBS is commonly used in cell culture as a source of nutrients and growth factors; however, it contains xenogeneic components that carry risks such as interspecies contamination, disease transmission, and regulatory and ethical concerns. Additionally, the composition of FBS is ill-defined, resulting in batch-to-batch variation and skewed experimental outcomes [51, 52].

**Figure 1:**
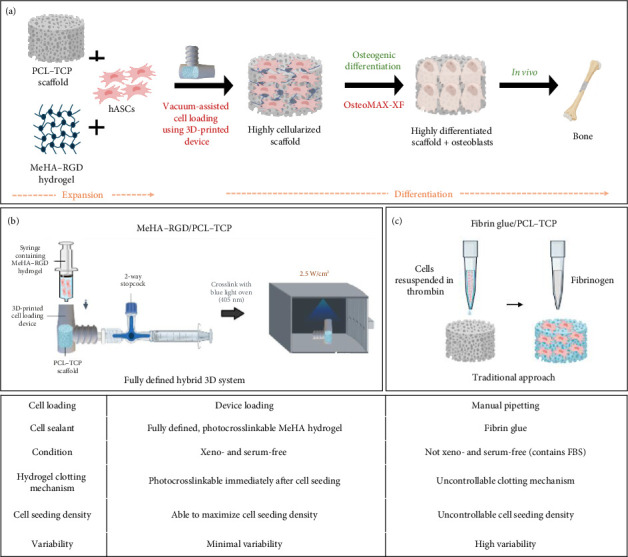
Schematic illustration of the cell loading process. (a) Overall procedure of hybrid scaffold with vacuum-assisted cell loading in a serum-free environment. (b) Device loading method of MeHA–RGD, (c) manual loading of two-component fibrin glue into PCL–TCP scaffold. PCL–TCP, polycaprolactone–tricalcium phosphate; MeHA, methacrylated hyaluronic acid; RGD, Arg-Gly-Asp; hASCs, human adipose-derived stem cells. Created with BioRender.com.

**Figure 2:**
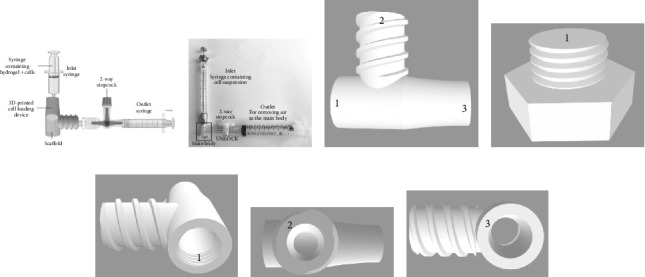
(a) Schematic representation of the vacuum-assisted cell loading device and method. The customized 3D-printed device snugly fits the scaffold and features an inlet and outlet with specialized Luer connections to establish a vacuum chamber. A hydrogel-cell suspension is introduced via the inlet syringe (labeled 3), while air is evacuated through the outlet syringe (labeled 2) to create a vacuum. The 2-way stopcock locks the vacuum, enabling the hydrogel to fill the scaffold. The loaded scaffold is photocrosslinked under 405 nm blue light. (b) Physical setup of device loading method (c) 3D design of the cell loading device. (d) Screw cap design. Inlets and outlets are labeled as follows: (e) 1-screw cap ensuring vacuum integrity, (f) 2-attachment point for the 2-way stopcock, and (g) 3-inlet syringe for cell seeding.

In this study, we devised a fully defined hybrid 3D system for BTE using a load-bearing osteogenic scaffold, seeded with human adipose-derived stem cells (hASCs) resuspended in a photocrosslinkable, fully defined hydrogel using a vacuum-assisted cell loading device (Figure 1(a)). We use a MeHA hydrogel, conjugated to an adhesion peptide (Arg-Gly-Asp, RGD), is introduced into the scaffold with low-pressure/vacuum employing a novel, custom-made 3D-printed cell loading device, promptly, and gently photocrosslinked at 405 nm to prevent cell leakage (Figures 1(b) and 2). Moreover, the entire process from in vitro stem cell expansion to osteogenic differentiation is carried out in fully defined, serum-free conditions. Eliminating animal and donor-derived components reduces variability in the BTE process, allowing standardization and reproducibility in a clinical setting. We subsequently evaluated the viability, adhesion, and osteogenic differentiation of the novel hybrid 3D system in comparison with the conventional manual loading using fibrin glue. We find that the fully defined cell-laden hydrogel loaded under vacuum can deliver at least comparable cell viability and osteogenic differentiation outcomes as the conventional fibrin glue method. Overall, our approach employs a consistent vacuum-assisted cell loading device to ensure efficient cell loading and eliminates the use of fibrin glue as well as any other nonhuman components.

## 2. Materials and Methods

### 2.1. Scaffold and Hydrogel Fabrication

A 3D-printed medical grade polycaprolactone (PCL)-20% TCP scaffold (Osteopore International, Singapore) was manufactured through fused deposition modeling (FDM) in a laydown pattern of 0/60/120°, featuring a 70% scaffold porosity to create a honeycomb-like pattern of triangular pores. Cylindrical-shaped scaffolds (Ø 5 mm × 6 mm) were extracted from the PCL–TCP sheets and underwent surface treatment with 5M sodium hydroxide (NaOH, Merck Millipore, USA) for 3 h to enhance their hydrophilicity. After three rinses with PBS, they were sterilized in 70% ethanol for 2 h, followed by additional rinsing with PBS and centrifugation to remove excess 70% ethanol. Subsequently, the scaffolds were placed in a 37°C incubator overnight for drying.

The preparation of fibrin glue was carried out using fibrinogen and thrombin (Sigma-Aldrich, USA) from bovine plasma, sterilized by a 0.2-μm filter membrane (Corning, USA), mixed at final concentrations of 25 mg/mL and 100 U/mL, respectively.

Photocrosslinkable MeHA (Advanced BioMatrix, USA) was dissolved in triethanolamine buffer (Sigma-Aldrich, USA) at a concentration of 2% (w/v). RGD peptide (GCGYGRGDSPG, MW 1025.06, Biomatik, Canada) dissolved in PBS was added to the MeHA solution, resulting in a final solution of 1 mM of RGD peptide, and the solution was allowed to react overnight at 37°C. The MeHA–RGD solution underwent dialysis against PBS with Slide-a-Lyzer dialysis cassettes (MWCO 20K, Thermo Scientific) as per the manufacturer's instructions. The dialyzed solution was then lyophilized using a freeze dryer (Labconco, FreeZone Freeze Dry Systems, USA). The lyophilized product was resuspended in PBS to a final concentration of 2% (w/v). The photocrosslinker lithium phenyl (2,4,6-trimethylbenzoyl) phosphinate (LAP) was added to MeHA at 0.02% and photocrosslinked with a blue light curing oven @ 405 nm (UVSZ-144A, UVET, China).

### 2.2. MeHA–RGD Characterization

The final dialyzed and lyophilized product of MeHA–RGD was characterized by ^1^H NMR to verify the conjugation of RGD peptide to the MeHA hydrogel. The MeHA–RGD sample was weighed and dissolved in H_2_O: D_2_O (1:9) at a final concentration of 2% (w/v) in a 1.5-mL Eppendorf tube. One-dimensional ^1^H-NMR spectra, with water signal suppression achieved by excitation sculpting, were recorded at 25°C on a Bruker Avance II 700 MHz NMR spectrometer equipped with a 5-mm z-gradient TXI cryoprobe using a standard Bruker pulse program (Bruker BioSpin GmbH, Germany).

### 2.3. Vacuum-Assisted Loading Using a 3D-Printed Cell Loading Device

The 3D-printed cell loading device and screw cap were designed using Autodesk Fusion 360 and fabricated via the PolyJet technique on an Objet500 Connex3 printer (Stratasys, Israel) with VeroClear material. The device chamber was customized to fit the scaffold dimensions (Ø 5 mm × 6 mm) used in this study.

**Figure 3:**
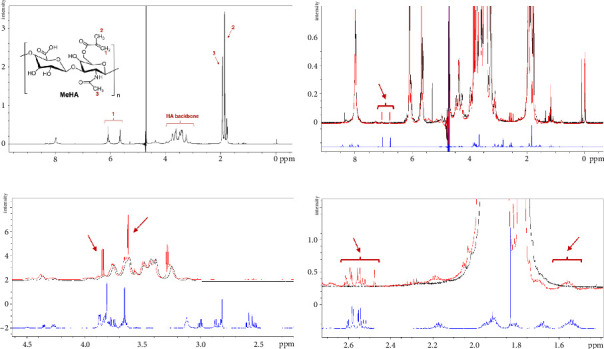
Conjugation of RGD peptide to MeHA hydrogel. (a) 1 H NMR spectra of MeHA, with Peak 1 representing the methylacrylate alkene (2 protons) and Peaks 2 and 3 representing the methyl group on methacrylate (3 protons) and HA (3 protons), respectively. (b)–(d) 1 H NMR spectra of MeHA (black), MeHA–RGD (red), and RGD peptide (blue) with overall spectra (b) and focused peaks (c) and (d). Specific peaks observed (indicated by red arrow) in MeHA–RGD spectra that are unique to RGD peptide spectra (b)–(d).

The device features a scaffold chamber positioned between an inlet and an outlet, both equipped with specific Luer connections (Figure 3). The scaffold was inserted through hole 1 and secured with a screw cap. A 100-μL SGE Gas Tight Syringe with Luer lock (Sigma-Aldrich, USA) served as the inlet syringe for hydrogel loading, while the outlet was connected to a 2-way stopcock and an empty 10-mL syringe to generate a vacuum.

For cell loading, the inlet syringe was filled with hASCs resuspended in a photocrosslinkable hydrogel (MeHA–RGD) and then attached to hole 3. Air was evacuated by pulling back the plunger of the outlet syringe, and the vacuum was maintained by securing the 2-way stopcock. The plunger of the inlet syringe was gradually depressed to evenly fill the scaffold with hydrogel-cell suspension. Once loaded, the device containing the scaffold was detached from the syringes and placed in a blue light curing oven (405 nm, UVET, China) for 15 s to photocrosslink the hydrogel. After photocrosslinking, the screw cap was removed, and the scaffold was carefully extracted from the device and immediately transferred to culture media.

### 2.4. Cell Culture and Seeding

hASCs were from StemPro Human Adipose Derived Stem Cell Kit (Thermo Fisher, USA). Initial expansion and cultivation of hASCs were performed in the MesenPRO RS medium, as recommended by the supplier. The expanded cells were cryopreserved for future use using NutriFreez D10 Cryopreservation Medium (Sartorius, Germany), an animal-component-free and serum-free freezing medium. In all experiments, cell culture flasks were precoated with MSC Attachment Solution (Sartorius, Germany), and cryopreserved hASCs were cultured to 70%–80% confluence in a xeno-free, serum-free MSC expansion medium, MSC NutriStem XF Medium (Sartorius, Germany) in a 37°C incubator with 5% CO_2_. hASCs from passages 2–5 were used for all experiments. Due to trypsin's animal-derived nature from bovine, porcine, and murine sources [53], we opted for a dissociation from the culture flask using the recombinant trypsin–EDTA solution (Sartorius, Germany), with the dissociation subsequently neutralized using soybean trypsin inhibitor (Biological Industries, Israel). Cells were resuspended in 100 μL of hydrogel (MeHA–RGD or fibrin glue) and seeded into 3D PCL–TCP scaffold (Ø 5 mm × 6 mm) at a concentration of 1 × 10^6^ cells/scaffold. The seeded scaffolds were then incubated overnight in MSC NutriStem XF growth medium.

### 2.5. MeHA–RGD and Fibrin Glue Loading Into PCL–TCP Scaffolds

For MeHA–RGD loading, cells were resuspended in MeHA–RGD hydrogel and seeded into the 3D scaffold using the vacuum-assisted cell loading device, followed by 15 s of blue light exposure. For fibrin glue loading, cells were resuspended in thrombin and seeded into the 3D scaffold, followed by the addition of an equal volume of fibrinogen. The fibrin glue-seeded scaffold was placed in a 37°C incubator with 5% CO_2_ for 1h before further incubation in growth media. The next day, the growth medium was replaced with a defined xeno-free and serum-free osteogenic differentiation medium, OsteoMAX-XF (Merck Millipore, USA), for 21 days. The culture medium was changed every 2-3 days.

### 2.6. Cell Metabolic Activity

Metabolic activity in the cells was assessed using the alamarBlue colorimetric assay (Invitrogen, Thermo Fisher Scientific) on Days 0, 7, 14, and 21. Optimal alamarBlue incubation duration for the cell seeding density employed in these experiments has been previously optimized (data not shown). The scaffolds were washed with PBS and were then immersed in a 10% alamarBlue solution for 30 min in a 37°C incubator with 5% CO_2_. Following incubation, the reduced alamarBlue solutions were transferred to a black, 96-well plate and measured for fluorescence at 560/590 nm using a microplate reader (Synergy H1, BioTek, USA). Results were normalized to Day 0 and expressed as fold change.

### 2.7. Cell Viability Staining

Cell viability was evaluated and visualized using calcein AM/ethidium homodimer-1 (EthD) staining with a live/dead viability kit (Invitrogen, Thermo Fisher Scientific), according to the manufacturer's instructions. The scaffolds were imaged using an inverted fluorescence live cell microscope (Axio Observer 7, Zeiss, Germany) and analyzed with ImageJ software.

### 2.8. Gene Expression Analysis

The gene expression of eight markers related to osteogenic differentiation was assessed through quantitative real-time polymerase chain reaction (qRT-PCR). RNA was extracted from scaffold samples on Days 0, 7, 14, and 21 using an RNA extraction kit (RNeasy Mini Kit, Qiagen, USA). The extracted RNA was then reversely transcribed to cDNA using the High-Capacity cDNA Reverse Transcription Kit (Applied Biosystem, Thermo Fisher Scientific) following the manufacturer's instructions. All RT-PCRs were conducted under identical conditions, including denaturation at 95°C for 15 s, and annealing and extension at 60°C for 1 min, for 40 cycles using the StepOnePlus Real-Time PCR System (Applied Biosystems, Thermo Fisher Scientific, USA). Melting curve analysis was performed to verify primer specificity. The relative gene expression of each gene of interest was calculated by normalizing to the housekeeping gene GAPDH and expressed in the 2^−ΔΔCT^ method relative to the expression of undifferentiated hASCs (Day 0) as a control. Each time point was assessed in triplicates. The primer sequences used for qRT-PCR amplifications are listed in Table 1.

### 2.9. Calcium Content

Calcium deposition by differentiated hASCs in the scaffold was observed using alizarin red staining (ARS), a method used for visualizing mineralization in bone tissue. ARS and quantification reagents from the Osteogenesis Quantitation Kit (Merck Millipore, USA) were used for ARS on scaffold samples on Days 7, 14, and 21. The scaffolds were washed with PBS, fixed with 4% paraformaldehyde for 15 min at room temperature, and then gently washed with deionized H2O five times. Subsequently, 1 mL/well of ARS solution was added for 20 min at room temperature. After thorough washing with deionized H2O to remove excess dye, the scaffolds were imaged with an M60 Routine stereo microscope (Leica Microsystems, Germany). For ARS quantification, the scaffolds were transferred to a 1.5-mL microcentrifuge tube and immersed in 10% acetic acid for 30 min with vigorous vortexing. The microcentrifuge tubes were heated at 85°C for 10 min, then cooled down on ice for 5 min, and finally centrifuged at 20, 000 × g for 15 min. The acetic acid solution was neutralized to pH 4.1 to 4.5 with 10% ammonium hydroxide and read at 405 nm using a microplate reader (Synergy H1, BioTek, USA).

### 2.10. Cytoskeletal and Immunofluorescence Staining

Cytoskeletal (for actin) and immunofluorescence staining (for osteocalcin and osteopontin) were performed at each time point on Days 0, 7, 14, and 21. The scaffolds were washed with PBS and fixed with 4% paraformaldehyde for 20 min at room temperature, followed by two washes with PBS. After washing, thin slices of scaffold from each section (top and bottom) were obtained for staining for each time point. The scaffold slices were permeabilized with 0.2% Triton X-100 (Sigma-Aldrich, USA) and then incubated in a blocking buffer solution containing 5% bovine serum albumin, 2% goat serum, and 0.3M glycine in PBS for 2 h. Primary antibodies targeting osteocalcin and osteopontin (1:100, Abcam, UK) were diluted in an antibody buffer solution with 0.05% Tween-20 and 1% BSA and incubated at 4°C overnight on a shaker. The following day, the secondary antibody conjugated with Alexa Fluor 647 (1:250, Thermo Fisher Scientific, USA) was added and incubated for 2 h at room temperature on a shaker. For visualizing cellular adhesion and morphology on Days 0, 7, 14, and 21, actin filaments were visualized using Phalloidin-iFluor 555 reagent (Abcam, UK) and Hoechst 33342 (Invitrogen, Thermo Fisher Scientific), following the manufacturer's instructions. The scaffold slices were immersed in Phalloidin-iFluor 555 (1:5000) for 40 min at room temperature. Finally, Hoechst 33342 was added into the solution and incubated for 10 min, followed by thorough washing with PBS. Images were acquired using an Inverted Live-Cell Confocal Laser Scanning Microscope (LSM 800, Carl Zeiss, Germany).

### 2.11. Statistical Analysis

Statistical analysis was performed using GraphPad Prism software (v9, USA) to conduct multiple unpaired Student's *t*-tests with Welch's correction for datasets with multiple data points. Results are presented as mean ± standard deviation (SD) with data considered statistically significant at ^∗^*p* < 0.05, ^∗∗^*p* < 0.01, ^∗∗∗^*p* < 0.001, ^∗∗∗∗^*p* < 0.0001, and ns = nonsignificant. Welch's correction was selected because the experimental groups (MeHA–RGD/PCL–TCP and fibrin glue/PCL–TCP) are unlikely to have equal variances between groups.

## 3. Results

### 3.1. Successful Conjugation of RGD Peptide to MeHA Hydrogel Confirmed by ^1^H NMR Analysis

^1^H NMR was conducted to characterize MeHA, RGD, and MeHA–RGD (Figure 3). The characteristic peaks of the methacrylate alkene group in MeHA at *δ* = 6.1, 5.7 ppm, and the methyl groups on methacrylate and HA at *δ* = 1.8–2.0 ppm are observable (Figure 3(a)). Specific peaks at *δ* = 6.7, 7.0 (Figure 3(b)), *δ* = 3.6, 3.8 (Figure 3(c)), and *δ* = 1.5–1.6, 2.5–2.6 ppm (Figure 3(d)) can be identified in the ^1^H NMR spectra of MeHA–RGD (red), which are unique to the RGD spectra (as indicated by the red arrow). The RGD peptide sequence, GCGYGRGDSPG, contains two special amino acids, cysteine and proline, and a hydrophobic amino acid, tyrosine. Characteristic peaks at *δ* = 6.7, 7.0 ppm (Figure 3(b)) and *δ* = 2.5–2.6 ppm (Figure 3(d)) in the ^1^H NMR spectra of MeHA–RGD corresponded to the aromatic structure of tyrosine and the thiol-containing group in cysteine, respectively. Peaks indicated at *δ* = 3.6 and 3.8 (Figure 3(c)) could correspond to the five-member nitrogen-containing ring structure in proline with characteristics peaks at *δ* = 2.0, 3.41, and 4.12.

The presence of these peaks in the ^1^H NMR spectra of MeHA–RGD and not MeHA confirms the successful conjugation of RGD peptide to the MeHA hydrogel.

### 3.2. Vacuum-Assisted Cell Loading Device Achieves Superior Efficiency in Cell Loading Without Compromising Osteogenic Differentiation

The vacuum-assisted cell loading device was compared to manual loading to assess cell viability, distribution, and osteogenic differentiation. Loading of cells using a manual pipette and the vacuum-assisted cell loading device was described as manual and device loading, respectively, from hereon. 3D-printed device was designed and customized to snugly fit the scaffold, with an inlet and outlet, featuring specialized Luer connection designs to establish a vacuum chamber for vacuum-assisted cell loading. Hydrogel-containing cells were introduced into the inlet syringe, which was then attached with a Luer connection as shown. Subsequently, the outlet syringe was drawn to eliminate air from the device until a suction was felt. Upon achieving desired vacuum, the 2-way stopcock is then locked to maintain vacuum within the apparatus. The plunger of the inlet syringe was pushed to fill the scaffold with hydrogel-containing cells. The device containing the cell-loaded scaffold was crosslinked with blue light oven at 405 nm (Figure 2).

**Figure 4:**
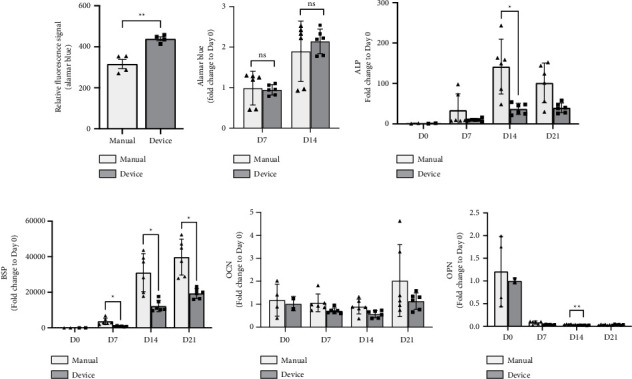
(a) Cell loading between manual and device loading was measured with alamarBlue assay (*n* = 4). (b) Cell metabolic activity of samples (*n* = 6, 3 scaffolds total) was measured with alamarBlue assay over 14 days, normalized to Day 0 (*n* = 3). Relative fold change determined by quantitative real-time PCR of osteogenic differentiation markers (c) alkaline phosphatase (ALP), (d) bone sialoprotein (BSP), (e) osteocalcin (OCN), and (f) osteopontin (OPN) (*n* = 4–6). All values are reported as mean ± SD, where symbols (^∗^*p* < 0.05, ^∗∗^*p* < 0.01, ^∗∗∗^*p* < 0.001, ^∗∗∗∗^*p* < 0.0001) indicate significance levels and ns = nonsignificant, based on unpaired Student's *t* tests with Welch's correction.

Cell loading efficiency was tested by seeding 1 million hASCs resuspended in the MeHA–RGD hydrogel into a PCL–TCP scaffold using manual and device loading, and it was measured with the alamarBlue assay. Results (Figure 4(a)) indicated significantly higher cell loading efficiency (*p* < 0.01) with a smaller SD using device loading. hASCs loaded into PCL–TCP scaffold via manual or device loading were cultured in osteogenic differentiation media for 14 days with alamarBlue measured on Days 0, 7, and 14 to assess the metabolic activity of hASCs (Figure 4(b)). Results from the alamarBlue assay were comparable with the manual loading method (ns), suggesting that the low pressure/vacuum in device loading did not negatively impact cell viability and metabolic activity. This trend was consistent at all time points. Interestingly, the device loading method displayed a smaller SD than manual loading as well. Osteogenic differentiation of hASCs loaded manually or with a device was characterized by RT-PCR over 21 days (Figures 4(c), 4(d), 4(e), and 4(f)). Both the manual and device loading groups exhibited increased ALP expression levels from Days 0–14 and Days 0–21, respectively (Figure 4(c)), suggesting ongoing osteogenic differentiation of hASCs in both loading methods. An increasing trend in BSP expression can be observed in both manual and device loading groups (Figure 4(d)). Both ALP and BSP exhibited a higher (*p* < 0.05) expression in manual loading compared to device loading on Days 14 and 21. Both groups showed a peak in OCN expression on Day 21 (Figure 4(e)), while there was no increase in OPN expression on Day 21 (Figure 4(f)). No significant difference in OCN and OPN expression was observed between manual and device loading. Overall, the osteogenic gene expression of hASCs in device loading was comparable to manual loading, indicating positive regulation of osteogenic differentiation in both groups. Hence, hASCs seeded via device loading can remain viable and proliferative and undergo osteogenic differentiation similarly to those loaded manually (Figure 4).

### 3.3. Hybrid MeHA–RGD/PCL–TCP Scaffold Enhances Metabolic Activity and Provides Uniform Cell Distribution Compared to Fibrin Glue/PCL–TCP

The fully defined hybrid 3D scaffold system integrates a photocrosslinkable MeHA–RGD hydrogel (characterized in Section 3.1) encapsulating cells with a load-bearing PCL–TCP scaffold, utilizing a vacuum-assisted cell loading approach. Following validation (Section 3.2), the performance of the hybrid 3D scaffold system was compared with fibrin glue, a conventionally used cell sealant, to evaluate differences in cell viability, metabolic activity, and osteogenic differentiation potential. The hybrid 3D scaffold system and fibrin glue loading were referred to as the MeHA–RGD/PCL–TCP and fibrin glue/PCL–TCP scaffolds, respectively.

**Figure 5:**
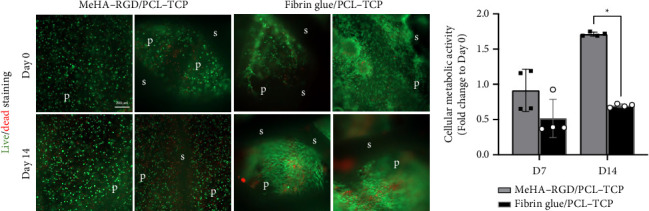
Comparison of cell viability and metabolic activity in MeHA–RGD/PCL–TCP scaffolds versus fibrin glue/PCL–TCP scaffolds in osteogenic differentiation medium. (a) Live/dead staining was used to assess cell viability on Days 0 and 14. Live cells are indicated in green, and dead cells in red. The scaffold structure includes struts (s) and pores (p), as labeled. Cells were cultured in osteogenic differentiation medium (OsteoMAX-XF) at 37°C with 5% CO_2._ Scale bar indicates 200 μm for all images. (b) Cellular metabolic activity was quantified using the alamarBlue assay up to Day 14 (*n* = 4), with results normalized to Day 0 and expressed as fold change. Cells were seeded at 1 million per scaffold and cultured under the same conditions. All values are reported as mean ± SD, where symbols (^∗^*p* < 0.05, ^∗∗^*p* < 0.01, ^∗∗∗^*p* < 0.001, ^∗∗∗∗^*p* < 0.0001) indicate significance levels, based on unpaired Student's *t* tests with Welch's correction.

MeHA–RGD and fibrin glue were loaded into the 3D-printed cylindrical PCL–TCP scaffold as described in Figure 1. The viability and metabolic activity of hASCs within the MeHA–RGD/PCL–TCP and fibrin glue/PCL–TCP scaffolds were assessed using live/dead staining and the alamarBlue assay, respectively, over 14-day culture period (Figure 5(a)). Distinct differences in the morphology of hASCs within the two scaffolds were observed. In the MeHA–RGD/PCL–TCP scaffold, cells appear rounded and evenly distributed within the scaffold, occupying both the pores and the struts. Conversely, hASCs within the fibrin glue/PCL–TCP scaffold exhibited an elongated morphology, often concentrated in specific regions of the scaffold. Despite these differences, live/dead staining confirmed high cell viability in both scaffolds after 14 days of culture. Metabolic activity, as measured by the alamarBlue assay, indicated significantly higher activity (^∗^*p* < 0.05) in the MeHA–RGD/PCL–TCP scaffold on Day 14 compared to the fibrin glue/PCL–TCP scaffold (Figure 5(b)). These results suggest that the MeHA–RGD hydrogel supports cell viability and metabolic function effectively, comparable to fibrin glue.

### 3.4. MeHA–RGD/PCL–TCP Scaffold Promotes Osteogenic Differentiation With Comparable Marker Expression to Fibrin Glue/PCL–TCP

**Figure 6:**
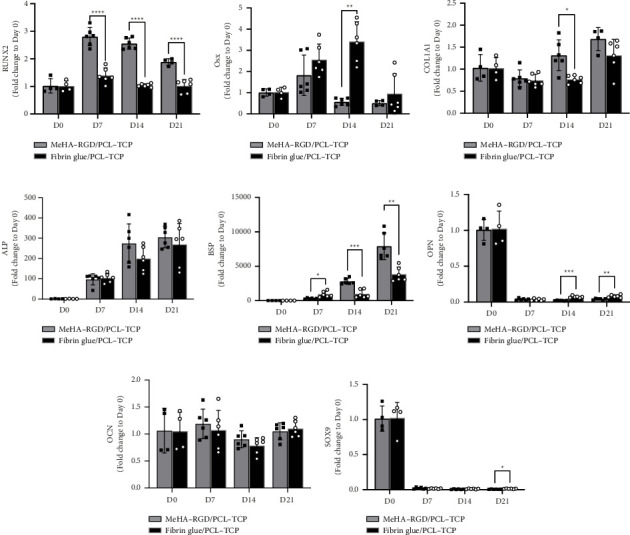
Real-time PCR analysis of osteogenic markers of hASCs differentiated in MeHA–RGD/PCL–TCP scaffold and fibrin glue/PCL–TCP scaffold (*n* = 4–6). Gene expression (fold change to Day 0) of osteogenic differentiation regulatory markers (a) and (b) early osteogenic differentiation markers (c)–(e), intermediate/late osteogenic markers (f)–(g), and chondrogenic differentiation marker (h) on Days 7, 14, and 21. RUNX2, runt-related transcription factor 2; Osx, osterix; COL1A1, collagen type 1 alpha 1; ALP, alkaline phosphatase; BSP, bone sialoprotein; OPN, osteopontin; OCN, osteocalcin; SOX9, SRY-box transcription factor 9. Cells were seeded at 1 million per scaffold and cultured in osteogenic differentiation medium (OsteoMAX-XF) at 37°C with 5% CO_2._ All values are reported as mean ± SD, where symbols (^∗^*p* < 0.05, ^∗∗^*p* < 0.01, ^∗∗∗^*p* < 0.001, ^∗∗∗∗^*p* < 0.0001) indicate significance levels, based on unpaired Student's *t* tests with Welch's correction.

To investigate the osteogenic differentiation potential of hASCs in the hybrid system, the expression of osteoblastic markers was analyzed using qRT-PCR (Figure 6). RUNX2 expression, increases during osteogenesis, peaked on Day 7 in both scaffolds. However, RUNX2 expression was significantly higher in the MeHA–RGD/PCL–TCP compared to the fibrin glue/PCL–TCP scaffold on Days 7 and 14 (^∗^*p* < 0.0001) and on Day 21 (^∗∗^*p* < 0.001) (Figure 6(a)). Osx expression peaked on Days 7 and 14 for MeHA–RGD/PCL–TCP scaffold and fibrin-glue/PCL–TCP scaffold, respectively, followed by downregulation (Figure 6(b)). Osx expression was significantly higher (^∗∗^*p* < 0.01) on Day 14 in the fibrin glue/PCL–TCP group compared to the MeHA–RGD/PCL–TCP scaffold. The expression of COL1A1, a marker of extracellular matrix production, was elevated in the MeHA–RGD/PCL–TCP scaffold, with a significant difference observed on Day 14 (^∗^*p*< 0.05). ALP expression increased consistently from Day 7 to Day 21 in both scaffolds, with no significant difference between them (Figure 6(d)). BSP expression showed an exponential increase in both scaffolds, with significantly higher levels in the MeHA–RGD/PCL–TCP group (Figure 6(e)). These findings suggest that early osteogenic markers, particularly ALP and BSP, were highly sensitive indicators in this study.

OPN expression increased from Day 14 to Day 21 in the MeHA–RGD/PCL–TCP scaffold from Day 7 to Day 21 in the fibrin glue/PCL–TCP groups. However, OPN levels were significantly higher in the fibrin glue/PCL–TCP scaffold on Days 14 (^∗∗∗^*p* < 0.001) and 21 (^∗∗^*p* < 0.01) (Figure 6(f)). OCN expression did not differ significantly between the two scaffolds (Figure 6(g)). SOX9, a chondrogenic marker, was significantly downregulated in both groups, indicating that hASCs were directed away from a chondrogenic lineage (Figure 6(h)). Overall, these findings confirm that both scaffolds support osteogenic differentiation, with MeHA–RGD/PCL–TCP demonstrating comparable or superior gene expression levels for key osteogenic markers.

**Figure 7:**
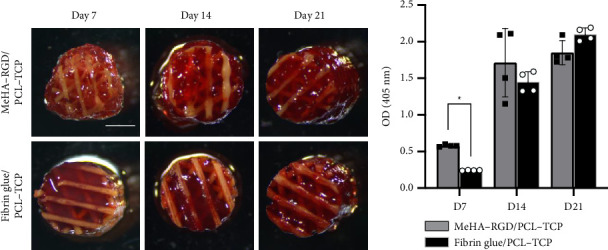
Alizarin red staining and quantification of calcium deposition in hASCs differentiated in MeHA–RGD/PCL–TCP scaffolds compared to fibrin glue/PCL–TCP scaffolds (*n* = 4). (a) Representative images of alizarin red staining illustrate calcium deposition within the scaffolds. The red staining intensity correlates with the level of mineralization. Cells were cultured in osteogenic differentiation medium (OsteoMAX-XF) at 37°C with 5% CO2, with medium changes every 2-3 days. Scale bar indicates 2 mm for all images. (b) Quantification of alizarin red staining was performed by measuring absorbance at 405 nm on Days 7, 14, and 21. Cells were seeded at 1 million per scaffold and cultured under the same conditions. Results are compared between scaffolds loaded using the device (MeHA–RGD/PCL–TCP) and those loaded manually (fibrin glue/PCL–TCP). All values are reported as mean ± SD, where symbols (^∗^*p* < 0.05, ^∗∗^*p* < 0.01, ^∗∗∗^*p* < 0.001, ^∗∗∗∗^*p* < 0.0001) indicate significance levels, based on unpaired Student's *t* tests with Welch's correction.

To assess the extent of mineralization, ARS was performed on hASCs cultured in MeHA–RGD/PCL–TCP and fibrin glue/PCL–TCP scaffolds for 21 days (Figure 7). ARS results showed a steady increase in calcium deposition over time, peaking on Day 21 in both groups (Figures 7(a) and 7(b)). On Day 7, the MeHA–RGD/PCL–TCP demonstrated significantly greater calcium deposition (^∗^*p* < 0.05) compared to fibrin glue/PCL–TCP scaffold (Figure 7(b)). These findings indicate that the MeHA–RGD/PCL–TCP scaffold effectively supports mineralization and osteogenic differentiation, achieving results comparable to the fibrin glue/PCL–TCP scaffold.

**Figure 8:**
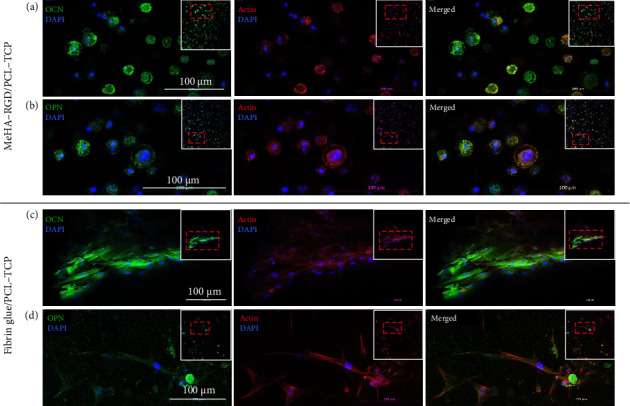
Immunofluorescence staining of differentiated hASCs. Osteocalcin (a) and (c) and osteopontin (b) and (d) were similarly expressed in both MeHA–RGD/PCL–TCP and Fibrin glue/PCL–TCP scaffolds after 21 days of osteogenic differentiation. Scale bar indicates 100 μm for all images.

The expression of OCN and OPN was further validated through immunofluorescence staining (Figure 8). Both markers were consistently expressed in hASCs cultures within the MeHA–RGD/PCL–TCP (Figures 8(a) and 8(b)) and fibrin glue/PCL–TCP (Figures 8(c) and 8(d)) scaffolds after 21 days of differentiation. Although morphological differences with hASCs seeded in MeHA–RGD appear rounded and evenly dispersed while those in fibrin glue were elongated and clustered, osteogenic differentiation was evident in both scaffolds. The results suggest that MeHA–RGD, combined with vacuum-assisted cell loading, offers a robust platform for osteogenic differentiation in 3D hybrid scaffolds. This photocrosslinkable, defined cell sealant represents a promising alternative to fibrin glue for load-bearing tissue engineering applications.

## 4. Discussion

The main objective of this study was to demonstrate that a novel, fully defined hybrid 3D system offers a compelling alternative to the current manual fibrin glue seeding technique for critical-sized 3D scaffolds, thus accelerating its clinical translation, particularly under serum- and xeno-free conditions.

Fibrin glue is a versatile material in tissue engineering, primarily used as a cell sealant to contain cells within scaffolds, though it can also be incorporated into bioinks. Pitacco et al. [54] formulated a bioink of fibrinogen, gelatin, hyaluronic acid, and glycerol, suspended with human bone marrow-derived mesenchymal stem cells (hBMSCs). This bioink was bioprinted into PCL frames to create mechanically reinforced fibrin structures. In a rat femur defect model, early hypertrophic constructs supported angiogenesis and bone regeneration with reduced ectopic bone formation, demonstrating that fibrin-based bioinks can facilitate in vitro cartilage formation and promote bone defect repair through early hBMSC hypertrophy. However, the poor mechanical properties of fibrin glue limit its suitability as a scaffold for critical-sized defects, making it commonly used as a cell sealant [55–58]. Derived from platelet-rich plasma (PRP), fibrin glue is biocompatible, biodegradable, and available in autologous or commercial xenogeneic forms [59]. While autologous fibrin glue minimizes immunogenic risks, its properties can vary significantly depending on patient-specific factors such as gender, age, and health status. Conversely, xenogeneic fibrin glue carries the potential risks of immunogenic reactions and transmission of viral or prion diseases [60].

Despite advances in tissue engineering applications, fibrin glue presents notable limitations that hinder its standardized clinical use. These include rapid shrinkage and degradation after polymerization, particularly when embedded with cells, as well as poor mechanical properties and batch-to-batch variability. Such issues affect handling, long-term stability, standardization, and reliability [50]. Therefore, to accelerate clinical translation, efforts should focus on developing fully defined components that ensure homogeneous cell distribution, efficient cell seeding, and robust support for cell viability and osteogenic differentiation within scaffolds.

Hyaluronic acid, an abundantly component of the extracellular matrix, plays a crucial role in many signaling pathways [61]. Use of recombinant hyaluronic acid eliminates the use of animal- and biologically derived components, minimizing variability. Methacrylation of hyaluronic acid results in a photocrosslinkable hydrogel with tunable properties [62]. To enhance cellular adhesion, we conjugated the cell-adhesive peptide RGD to MeHA, forming the MeHA–RGD hydrogel.

In recent years, photocrosslinkable MeHA hydrogels have gained traction in bone regeneration applications, highlighting its potential as a promising strategy for encapsulating cells in 3D scaffolds for bone repair [63–66]. For instance, a hydrogel formed from collagen and hyaluronic acid via photocrosslinking was used to encapsulate BM-MSCs, enhancing cell adhesion, proliferation, and osteogenic differentiation [63]. Another study produced a microgel combining photocrosslinkable gelatin methacrylate (GelMA) and MeHA, along with porcine decalcified bone matric (DBM) and vascular endothelial growth factor (VEGF), effectively promoting cell adhesion, proliferation, and osteogenic differentiation of BM-MSCs in vitro [65]. Additionally, Patel and Koh demonstrated that ASCs incorporated into a composite hydrogel of photocrosslinkable MeHA and PCL nanofibers supported cell adhesion and the expression of osteogenic markers such as COL1, ALP, and RUNX2, along with positive ARS [66]. The morphology of ASCs reported in this study mirrored those observed in our research (Figure 6), with comparable expression patterns of COL1, ALP, and RUNX2. These consistent results affirm that our functionalized MeHA–RGD hydrogel provides a suitable microenvironment for BTE, aligning with previous findings and demonstrating its efficacy.

Achieving high cell seeding efficiency is crucial for successful bone regeneration. Higher cell numbers lead to improved scaffold colonization, increased cell proliferation, and osteogenic differentiation [67–69]. This is particularly important in addressing critical-sized defects, as a uniform cell distribution with high cell seeding efficiency within the scaffold is necessary for creating constructs with homogeneous properties. Previous studies have reported on cells seeded into 3D porous PCL–TCP scaffold with various cell seeding density [70–73]. Notably, the cell seeding densities in three of these studies were relatively low given the scaffold size. Only Kurzyk et al. [71] compared three different cell seeding densities, finding the highest proliferation in the 0.9 × 10^6^ cells group after 7 days of culture in PCL–TCP scaffolds, compared to the 0.5 × 10^6^ and 1.5 × 10^6^ cells groups. This suggests an optimal seeding density of 0.9 × 10^6^ cells per scaffold. Given that our scaffold dimensions are comparable to those in Kurzyk's study, and based on our preliminary studies (not shown), we selected a seeding density of 1 × 10^6^ cells per scaffold.

Various cell seeding methods have been reported, ranging from static culture and pipetting, to syringe and low-pressure-assisted systems [67]. In our study, we demonstrate a straightforward and consistent vacuum-assisted cell loading system to efficiently load cells into the scaffold. We first fabricated a fully defined photocrosslinkable MeHA–RGD hydrogel by conjugating the adhesive peptide RGD to MeHA, characterized by ^1^H-NMR. RGD is a well-known ligand that enhances cell adhesion and spreading, thereby influencing cell behavior and function [74, 75]. Preliminary studies (not shown) compared two adhesion peptides, including RGD and DGEA. The MeHA hydrogel functionalized with the RGD peptide demonstrated significantly higher cell viability compared to MeHA alone and MeHA functionalized with the DGEA peptide. These findings align with existing literature, which reports improved cell adhesion when incorporating the RGD peptide, but not the DGEA peptide [76, 77]. Characterization using ^1^H-NMR (Figure 4) revealed unique RGD peaks in MeHA–RGD hydrogel, confirming the successful conjugation of the RGD peptide to the MeHA hydrogel. This MeHA–RGD hydrogel can be photocrosslinked at 405 nm immediately following cell seeding, minimizing the potential for cell leakage from the scaffold. Next, we validated the impact of low pressure/vacuum environment on the viability, proliferation, and osteogenic differentiation capability of hASCs (Figure 5). To make this comparison, cells resuspended in MeHA–RGD hydrogel were loaded into the scaffold using manual pipetting and the 3D-printed, vacuum-assisted cell loading device.

Previous studies have explored various vacuum-assisted cell seeding techniques, demonstrating the safety and reproducibility of the approach with uniform cell distribution in different models [55, 67]. A study by Solchaga et al. [55] presented a rapid seeding technique for large cell/scaffold composite constructs. This method utilized a SpeedVac rotary evaporator as the vacuum chamber, connected to a vacuum pump and gauge. Scaffolds were placed in a multiwell plate, covered with cell suspensions, and subjected to six cycles of vacuum and rapid release to atmospheric pressure over 10 min to remove trapped air. Another study [67] compared four cell seeding techniques for BM-MSCs into β-TCP scaffolds: soak, low-pressure, pipette, and syringe systems. The low-pressure system reported in the study [67] is similar to the previous study conducted by Solchaga et al. The pipette system is the traditional manual pipetting method, involving pipetting growth media containing cells directly into scaffolds and placing them in growth media. The syringe system involves soaking the scaffolds in a 50-mL syringe with growth media alone, then pulling the syringe with a pressure of 100 kPa and vibrating to remove bubbles from the scaffold. The process is repeated multiple times for 100 s, and the scaffold is then soaked in a cell suspension with media for 2 h. Among these, the syringe system demonstrated the highest cell seeding efficiency, leading to superior osteogenic differentiation in vitro and bone formation in vivo.

We developed a fully defined hybrid 3D system by integrating a MeHA–RGD/PCL–TCP hybrid scaffold with hASCs using a vacuum-assisted cell loading device. The entire process was conducted under sterile conditions, completely free of serum and xenogeneic components. Cells were expanded and differentiated in a serum- and xeno-free medium, with cryopreservation media also devoid of serum and xenogeneic elements. We replaced animal-derived trypsin with recombinant trypsin, neutralized with a soybean trypsin inhibitor, and used biotechnologically produced, animal-free recombinant MeHA. We sought to minimize potential variations, ensuring a reproducible and clinically relevant approach for BTE.

In the present study, we present a simple, rapid vacuum-assisted technique using a customizable 3D-printed cell loading device, photocrosslinkable MeHA–RGD hydrogel, and readily available laboratory materials, such as syringes. In comparison, our MeHA–RGD/PCL–TCP hybrid scaffold addresses several limitations of fibrin glue. By using recombinant hyaluronic acid and eliminating serum and xeno-derived components, we ensured consistency and reduced variability. Moreover, our vacuum-assisted cell loading technique, combined with the photocrosslinkable MeHA–RGD hydrogel, provides a robust and reproducible method for homogeneous cell distribution within scaffolds. The photocrosslinked hydrogel prevents cell leakage, controlled seeding, and facilitates handling, addressing critical issues associated with fibrin glue. Validation of the cell loading device showed higher cell seeding efficiency than manual pipetting, with sustained cell viability observed over 14 days. Additionally, cells loaded with the device successfully underwent osteogenic differentiation in the scaffold, expressing osteogenic-related markers, such as ALP, BSP, and OCN. Furthermore, cells loaded alternatively by pipetting, or our device exhibited similar trends in terms of cell viability and expression of osteogenic-related markers, indicating that the low pressure/vacuum environment did not affect cell viability and the osteogenic differentiation of hASCs. Moreover, the hydrogel in our study can be promptly and gently photocrosslinked after cell seeding and placed directly in osteogenic media. This method eliminates the need for complex machinery and lengthy incubation periods, offering a straightforward and efficient method of cell seeding into scaffolds.

Next, cell viability and osteogenic differentiation, which are key outcomes for successful tissue engineering, were directly compared between MeHA–RGD/PCL–TCP and fibrin glue/PCL–TCP scaffolds. Live/dead staining and alamarBlue assays revealed sustained cell viability over 14 days in both scaffolds. However, live/dead staining revealed distinct differences in cell morphology and distribution between the two groups. In the MeHA–RGD/PCL–TCP group, cells appeared rounded and evenly distributed throughout the scaffold. In contrast, in the fibrin glue/PCL–TCP group, cells were elongated and clustered in certain regions, leading to irregular cell distribution (Figures 5(a) and 8). This observation is consistent with other studies employing vacuum-assisted cell seeding techniques, which also reported uniform cell distribution [55, 67]. Interestingly, the results of the alamarBlue assay (Figure 5(b)) exhibited a smaller SD in the MeHA–RGD/PCL–TCP group compared to the fibrin glue/PCL–TCP group. This suggests that the vacuum-assisted device resulted in standardized cell seeding, resulting in higher consistency and reproducibility, which is crucial for facilitating translation to clinical settings.

We further assessed in vitro osteogenic differentiation of hASCs within the hybrid 3D scaffold system using qRT-PCR, ARS, and immunofluorescence staining. The MeHA–RGD/PCL–TCP scaffold effectively promoted osteogenesis in hASCs, evidenced by an increased expression of osteogenic markers RUNX2, Osx, ALP, and BSP (Figure 6), significant calcium deposition (Figure 7), and positive staining for bone matrix proteins OCN and OPN (Figure 8). These findings are consistent with previous studies that reported enhanced osteogenic marker expression (RUNX2, ALP, and BSP) during hASC osteogenic differentiation [78]. The observed expression patterns of COL1, RUNX2, and ALP in MeHA-PCL composite hydrogels corroborate our findings [66].

RUNX2 and Osx are key regulators of osteogenesis. RUNX2 regulates the expression of downstream osteoblastic differentiation genes, such as COL1A1, OCN, and BSP [79, 80]; induces MSCs differentiation into immature osteoblasts [81]; and regulates Osx. ALP, a marker of mineralization, is highly expressed in cells of mineralized tissue and is an important biochemical marker of bone formation [82, 83]. BSP, an early marker of osteogenic differentiation [84], indicates a late stage of osteoblastic differentiation and an early stage of matrix mineralization [85]. Both scaffolds showed increasing trends in ALP and BSP expression.

OPN, an early-to-intermediate marker of osteogenic differentiation [86], was abundantly expressed on Day 0 due to secretion by MSCs [87]. Downregulation of SOX, a key chondrogenic marker [88], in both groups confirmed a shift away from chondrogenic lineage. During the osteogenic differentiation process from hASCs into mature osteoblasts or osteocytes, osteoblasts release and deposit calcium onto the surface [89] and start the mineralization process, which is a highly regulated physiological process in bones [90]. ARS serves to detect the presence and extent of mineralization resulting from calcium deposition as hASCs undergo differentiation into mature osteoblasts [89]. ARS revealed extensive calcium deposition in both groups on Day 21, with quantification supporting this observation. While qRT-PCR did not show significant increases in OCN and OPN expression, immunofluorescence staining on Day 21 of osteogenic differentiation confirmed their presence, suggesting their functional role in the matrix formation. Collectively, our results highlight the potential of the MeHA–RGD/PCL–TCP scaffold in supporting bone regeneration, offering promise for applications in tissue engineering and regenerative medicine.

While the presented hybrid system shows significant potential, several limitations need to be addressed before clinical translation. First, this study is limited to in vitro evaluations, which, although promising, do not fully replicate the complex in vivo microenvironment. The performance of the MeHA–RGD/PCL–TCP hybrid scaffold should be further validated in animal models with critical-sized bone defects to assess its efficacy and safety under physiological conditions. Second, the scalability of the vacuum-assisted cell seeding device for larger or irregularly shaped scaffolds was not explored in this study. A key advantage of this device is its ability to be customized based on defect size, making it a highly adaptable solution. However, future studies should evaluate whether the system's efficiency and uniformity are maintained with larger constructs. Third, regulatory constraints remain a significant challenge in translating BTE research into clinical applications. Therefore, it is crucial for research efforts to be oriented toward clinical translation from the outset, adopting a “bedside to bench and back again” approach [15]. Finally, further research should also focus on optimizing hydrogel functionalization to better mimic native bone tissue and enhance long-term regenerative outcomes. Addressing these limitations will be crucial in advancing this hybrid system toward preclinical and clinical studies, paving the way for effective scaffold-based regenerative therapies.

## 5. Conclusions

Our research demonstrates the potential and feasibility of a fully defined, serum-, and xenogeneic-free approach to BTE. A 3D-printed vacuum-assisted cell loading device within a hybrid 3D scaffold system was used in order to successfully address key limitations of existing methodologies, such as reliance on FBS and other nondefined components (such as fibrin glue). Our findings highlight the significant strides this approach makes toward standardization and reproducibility, which are critical for clinical translation. There is a notable scarcity of studies demonstrating in vitro culture of cells in 3D scaffolds under serum- and xeno-free conditions. To our knowledge, this is the first study to employ a 3D-printed vacuum-assisted cell loading device system within a fully defined hybrid 3D system under complete serum- and xeno-free conditions, with a focus on advancing standardization and reproducibility for future clinical translation.

Our results emphasize the ability to maintain robust cell viability and function in vitro under fully defined conditions, the successful integration of advanced manufacturing techniques such as 3D printing for scaffold preparation, and the demonstrated compatibility of these technologies for potential future applications in patient-specific therapies. While our approach has been effective in vitro, further in vivo studies are necessary to validate the hybrid 3D system for clinical application.

For future research, consideration of the following important steps is required: (1) In vivo testing: It is necessary to evaluate the performance of the hybrid scaffold and cell culture system in animal models to assess its regenerative capacity and integration with host tissues; (2) optimization of scaffold design: the 3D-printed cell loading device is customizable and can be optimized to enhance biomechanical properties specifically required for different bones. It is also scalable based on the size of the defect, offering the advantage of homogeneous cell distribution in a larger scaffold. This advancement is crucial for addressing critical-sized defects in clinical applications; and (3) regulatory considerations: addressing the regulatory requirements for clinical translation, including scalability, long-term safety evaluations, and compliance with regulatory requirements. Aligning research efforts with clinical translation from the outset will be key to accelerating the pathway toward clinical applications.

Overall, our research bridged the gap between laboratory research and clinical practice and demonstrated important foundational data necessary for the advancement of customizable xeno-free BTE strategies that can be effectively and safely translated to patient care in the future.

## Figures and Tables

**Table 1:** Primers used for qRT-PCR in this study.

Gene	Forward sequence	Reverse sequence
RUNX2	CTACCAGCCACCGAGACCAA	GTCACTGTGCTGAAGAGGCTG
Osterix (Osx)	CCTCTGCGGGACTCAACAAC	AGCCCATTAGTGCTTGTAAAGG
COL1A1	GGCAGCCCTGGTGAAAATGG	CCGGCAGCACCAGTAGCA
ALP	CCCGCTATCCTGGCTCCGTG	GCAATCGACGTGGGTGGGAG
OCN	AGGGCAGCGAGGTAGTGAA	TCCTGAAAGCCGATGTGGT
OPN	AACGCCGACCAAGGAAAACT	GGCCACAGCATCTGGGTATT
BSP	ATCTGTGCCACTCACTGCCTT	AGGCCATTCCCAAAATGCTGA
SOX9	CAGTACCCGCACTTGCACAAC	CTGCCCGTTCTTCACCGACTT

## Data Availability

The data presented in this study are included in this published article. Data and materials are available from the corresponding author subject to reasonable request and subject to the ethical approvals in place and material transfer agreements.
